# B Cells in Autoimmune Diseases

**DOI:** 10.6064/2012/215308

**Published:** 2012-12-12

**Authors:** Christiane S. Hampe

**Affiliations:** Department of Medicine, University of Washington, SLU-276, 850 Republican, Seattle, WA 98109, USA

## Abstract

The role of B cells in autoimmune diseases involves different cellular functions, including the well-established secretion of autoantibodies, autoantigen presentation and ensuing reciprocal interactions with T cells, secretion of inflammatory cytokines, and the generation of ectopic germinal centers. Through these mechanisms B cells are involved both in autoimmune diseases that are traditionally viewed as antibody mediated and also in autoimmune diseases that are commonly classified as T cell mediated. This new understanding of the role of B cells opened up novel therapeutic options for the treatment of autoimmune diseases. This paper includes an overview of the different functions of B cells in autoimmunity; the involvement of B cells in systemic lupus erythematosus, rheumatoid arthritis, and type 1 diabetes; and current B-cell-based therapeutic treatments. We conclude with a discussion of novel therapies aimed at the selective targeting of pathogenic B cells.

## 1. Introduction

Traditionally, autoimmune disorders were classified as T cell mediated or autoantibody mediated. However the improved understanding of the complexity of the immune system has significantly influenced the way we view autoimmune diseases and their pathogeneses. Reciprocal roles of T-cell help for B cells during adaptive immune responses and B-cell help in CD4+ T-cell activation are being increasingly recognized. The observation that most autoantibodies in traditionally autoantibody-mediated diseases are of the IgG isotype and carry somatic mutations strongly suggests T-cell help in the autoimmune B-cell response. Likewise B cells function as crucial antigen presenting cells in autoimmune diseases that are traditionally viewed as T cell mediated. This paper will discuss the role of B cells in autoimmune diseases; however, it needs to be emphasized that most autoimmune diseases are driven by a dysfunction in the immune network consisting of B cells, T cells, and other immune cells.

## 2. B-Cell Functions in Autoimmunity

**Figure 1:**
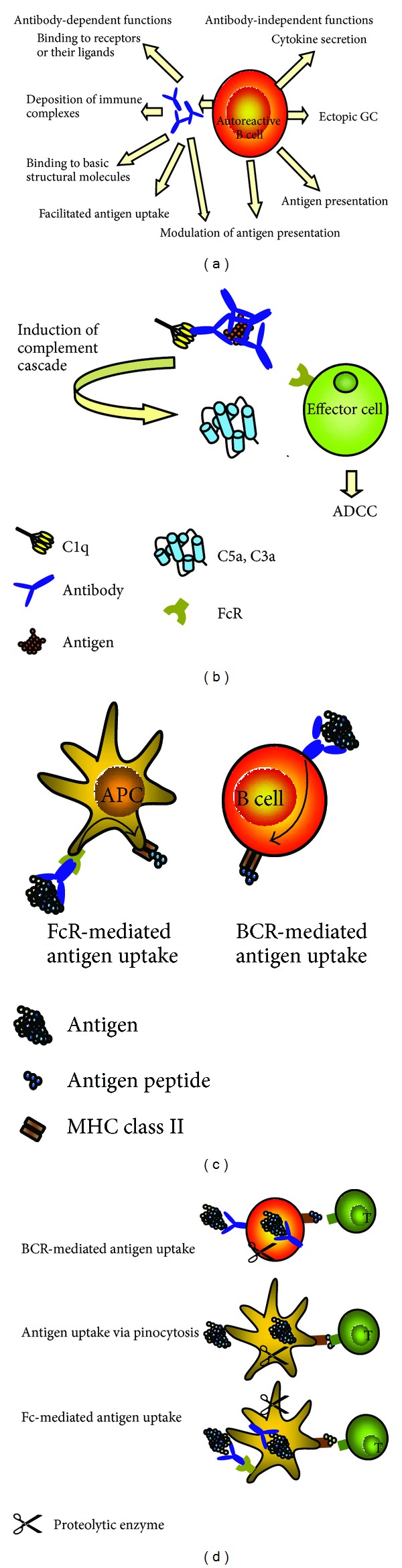
(a) B cells in autoimmune diseases. B cells have antibody-dependent and antibody-independent pathogenic functions. Secreted autoantibodies specific to receptors or receptor ligands can activate or inhibit receptor functions. Deposited immune complexes can activate complement and effector cells. Autoantibodies can bind to basic structural molecules and interfere with the synthesis of structural elements and facilitate the uptake of antigen. Independent of antibody secretion B cells secrete proinflammatory cytokines, support the formation of ectopic GCs, and serve as antigen presenting cells. Both secreted autoantibodies and BCR on B cells can modulate the processing and presentation of antigen and thereby affect the nature of presented T-cell determinants. (b) Pathogenic effects of deposited immune complexes. The Fc portion of antibodies in immune complexes can be bound by C1q of the classical complement pathway, which eventually leads to the release of C5a and C3a. These anaphylatoxins promote release of proinflammatory cytokines and serve as chemoattractants for effector cells. Moreover they induce the upregulation of activating FcR on effector cells. Binding of the Fc portion of the antibodies to FcR leads to activation of effector cells and further release of proinflammatory cytokines and proteolytic enzymes, mediators of antibody-dependent cell-mediated cytotoxicity (ADCC). (c) Effect of antibodies and antigen-specific B cells on antigen uptake. Left panel: antigen bound by antibody is taken up via FcR on APCs such as dendritic cells or macrophages. After processing, antigen is presented on MHC molecules. This FcR-mediated antigen uptake is more efficient than antigen uptake by pinocytosis. Right panel: antigen binds to the BCR of antigen-specific B cells and is internalized. B cells are highly efficient APCs in situations of low antigen concentrations. (d) Effect of antibodies and antigen-specific B cells on antigen processing and presentation. BCR-mediated antigen uptake can influence antigen processing and the nature of MHC-displayed T-cell determinants. Likewise, antigen/antibody complexes are bound by the FcR of APCs and processed in a unique fashion dependent on the epitope specificity of the bound antibody. The BCR or antibody can shield certain protein determinants from the proteolytic attack in endocytic compartments (represented as scissors in this figure). Presentation of some determinants may thereby be suppressed, while others are boosted. Thereby cryptic pathogenic peptides may be presented and stimulate autoreactive T cells.

Different functions of B cells can contribute to autoimmune diseases (Figure 1):

1. secretion of autoantibodies;
2. presentation of autoantigen;
3. secretion of inflammatory cytokines;
4. modulation of antigen processing and presentation;
5. generation of ectopic GCs.

These functions will be discussed in detail below.

### 2.1. Autoantibodies in Autoimmune Diseases

Autoantibodies can be detected in many autoimmune diseases. Their presence in the peripheral circulation and relative ease of detection makes them preferred markers to aid in diagnosis and prediction of autoimmune disorders. In some autoimmune diseases, the autoantibodies themselves have a pathogenic effect, as will be discussed in the following.

#### 2.1.1. Deposition of Immune Complexes and Inflammation (Figure 1(b))

The deposition of immune complexes composed of autoantibodies and autoantigens is a prominent feature of several autoimmune diseases, including systemic lupus erythematosus, cryoglobulinemia, rheumatoid arthritis, scleroderma, and Sjögren's syndrome. The immune complexes can trigger inflammation through activation of complement and Fc-receptor-dependent effector functions [15]. In the classical complement cascade, the Fc portion of the antibody is bound by complement component C1q, which eventually triggers the activation of the anaphylatoxins C5a and C3a. C5a and to a lesser degree C3a attract effector cells such as neutrophils and NK cells and stimulate the release of proteolytic enzymes and inflammatory cytokines. Activation of complement has been consistently demonstrated in experimental models of immune-complex diseases and in the kidneys of patients with systemic lupus erythematosus and lupus nephritis [16]. The immune complexes can also directly bind to Fc-receptors on effector cells leading to antibody-dependent-cell-mediated cytotoxicity (ADCC). 

#### 2.1.2. Stimulation and Inhibition of Receptor Function

Autoantibodies can affect receptor function with different outcomes as illustrated by autoantibodies targeting the thyroid stimulating hormone (TSH) receptor. TSH receptor autoantibodies in Graves' disease stimulate receptor function, triggering the release of thyroid hormones and development of hyperthyroidism [17], while TSH receptor autoantibodies in autoimmune hypothyroidism block the binding of TSH to the receptor [18]. Inhibitory autoantibodies are also found in Myasthenia gravis, where autoantibodies bind to the nicotine ACh receptors (AChRs) and block neurotransmission at the neuromuscular junction, inducing symptoms such as muscle weakness and fatigue [19], and in multifocal motor neuropathy, where autoantibodies bind to the ganglioside GM1 and cause motor neuropathy with conduction block at multiple sites [20]. Other autoantibodies can bind receptor ligands, preventing their binding to the receptor, as seen in Graves' disease with anti-TSH autoantibodies [21].  Table 1 summarizes other examples of receptor autoantibodies, their targets, pathogenic mechanisms, and associated diseases. 

#### 2.1.3. Facilitation of Antigen Uptake (Figure 1(c))

Autoantibodies facilitate antigen uptake by antigen presenting cells (APCs). Antigen complexed with antibodies is taken up via Fc receptors (FcRs) present on monocytes and dendritic cells [22]. This mechanism is more efficient than pinocytosis and results in 10–100-fold lower necessary antigen concentration for successful T-cell stimulation [23–26]. The importance of this mechanism has been demonstrated in a number of animal studies, where antibodies to various antigens enhanced T-cell responses to the respective antigens [27–29]. Autoantibodies can therefore break tolerance of normal T cells through their capacity to promote uptake of self-antigen by APCs via their FcRs. Indeed, autoantibodies to thyroid self-antigens dramatically enhanced uptake of thyroid peroxidase (TPO) by APCs and subsequent activation of TPO-reactive T cells [30] and blockade of Fc*γ*R markedly reduced this response [31]. Autoantibodies have also been demonstrated to facilitate the uptake of myelin by macrophages, and the removal of the Fc-portion of the antibodies prevented antigen uptake [32]. Moreover, Fc*γ*R–deficient DBA/1 mice were protected from myelin oligodendrocyte glycoprotein-induced experimental autoimmune encephalomyelitis (EAE), suggesting that FcR-mediated uptake of antibody-bound myelin is involved in the pathogenesis of multiple sclerosis [33]. Autoantibody-mediated antigen uptake may therefore be a critical mechanism in the pathogenesis of T-cell-mediated autoimmune diseases.

Further support for autoantibody-mediated antigen uptake as a pathogenic mechanism in autoimmunity comes from an elegant study by Harbers et al. where transgenic mice expressed ovalbumin (OVA) as “self” in both their thymus and pancreatic beta cells [34]. Presentation of OVA by dendritic cells to diabetogenic CD8+ OVA-reactive T cells was significantly stimulated by administration of antibodies specific to OVA. This response was not observed in mice lacking activating Fc*γ*R, indicating that the antibody-driven effector T-cell activation was indeed Fc*γ*R dependent.

However, autoantibodies are not always damaging to the organism, but can have protective functions [35, 36], and natural autoantibodies are commonly found in healthy individuals. Most of these antibodies are of the IgM isotype and have been speculated to have protective functions. One of these functions is the clearance of dying and aging cells and in mice natural IgM autoantibodies bind to epitopes specifically expressed on apoptotic cells [37, 38] enhancing the clearance of these cells, which may otherwise elicit a pathogenic autoimmune response [39, 40]. Lack of secreted IgM has been shown to correlate with an increase in pathogenic IgG autoantibodies and autoimmune disease possibly due to the lack of removal of apoptotic cells [41–43].

The mouse natural autoantibodies that arise without external antigen exposure are secreted from a subset of B cells, named B1 cells [44, 45], and a similar B-cell subset has been recently identified in humans [46]. In patients with SLE, higher levels of IgM associated with apoptotic cell clearance correlate with lower disease activity [47, 48], and healthy twins of SLE patients often present higher levels of these autoantibodies [49]. Another mechanism of protection by natural autoantibodies is the blockage of pathogenic autoantibodies to react with self-antigen [50], and titers of natural IgM specific to dsDNA correlated inversely with the severity of glomerulonephritis (GN) in SLE [51, 52]. 

Besides producing antibodies, activated B cells are also fundamental for coordinating T-cell functions as B-cell-depleted mice exhibit a dramatic decrease in numbers of CD4+ and CD8+ T cells, and a significant inhibition of memory CD8+ T cells [53, 54]. There are several antibody-independent mechanisms by which B cells can affect T cells and other immune cells as will be discussed below.

### 2.2. B Cells as Antigen-Presenting Cells

Especially at low antigen concentrations B cells function as superior APCs [55]. Other APCs (macrophages and dendritic cells) internalize antigen through pinocytosis, while B cells capture antigen through their antigen-specific B-cell receptors (BCRs) (Figure 1(c)). The ability of antigen-specific B cells to serve as efficient APCs has been demonstrated in several *in vivo* studies [56]. This mechanism is 1,000–10,000-fold more efficient than pinocytosis, and antigens can be successfully presented at very low concentrations, as those present in autoimmune diseases [57–59]. Moreover, the BCR-conferred antigen-specificity enables the B cells to focus the immune response to a specific antigen [60].

B cells serve as APCs in autoimmune diseases including rheumatoid arthritis and type 1 diabetes [61, 62]. Immunoglobulin-deficient mice in a model of autoimmune arthritis (proteoglycan-induced arthritis) did not develop arthritis. The observation that T cells isolated from proteoglycan-immunized transgenic mice that express membrane Ig (mIgM), but lack circulating antibodies, were unable to transfer disease suggested that these T cells were not adequately primed and that antigen-specific B cells may be required for this process. This was confirmed when direct targeting of proteoglycan to the BCR induced T cells competent to transfer arthritis [61].

The role of B cells as APC in type 1 diabetes is discussed in a separate chapter below.

### 2.3. Proinflammatory Cytokine Secretion

Activated B cells can secrete proinflammatory cytokines like interleukin-6 (IL-6), interferon-gamma (IFN-*γ*), IL-4, and TGF-beta [63–65]. These inflammatory mediators modulate the migration of dendritic cells, activate macrophages, exert a regulatory role on T-cell functions, and provide feedback stimulatory signals for further B-cell activation.

### 2.4. Modulation of Antigen Processing and Presentation

Besides facilitating antigen uptake, both membrane-bound and soluble antibodies can modulate the processing pattern of the antigen [66–69] (Figure 1(d)). Depending on the antigenic epitope recognized by the antibody or the BCR of the B cell, different T-cell determinants are presented on the MHC molecule [67, 70–73]. Indeed proteolysis of antigen-antibody complexes yielded protein fragments that were not observed in the absence of antibody [74]. This might have consequences for the ensuing T-cell response, in particular when otherwise cryptic T-cell determinants are presented. This bias in processing of antigen complexed with antibody may stem from antibody-mediated protection of distinct peptide sequences from degradation and/or sequestering of peptide sequences and interference with the loading of peptides onto MHC molecules [75]. 

The relevance of this mechanism in autoimmune diseases was suggested by studies showing that antibodies to thyroglobulin could augment or suppress processing and presentation of pathogenic T-cell determinants [76] and will be discussed further in the T1D chapter.

### 2.5. Ectopic Germinal Centers

B cells aid in the *de novo* generation of ectopic germinal centers (GCs) within inflamed tissues that can be observed during periods of chronic inflammation [77]. These ectopic structures are probably not a unique disease-specific occurrence, but a consequence of chronic inflammation. Activated T and B cells that infiltrate the site of chronic inflammation express membrane-bound lymphotoxin *α*_1_ *β*_2_ (LT*α*_1_ *β*_2_) [78]. High levels of LT*α*_1_ *β*_2_ eventually promote the differentiation of resident stromal cells into follicular dendritic cells (FDCs) and the development of ectopic GCs [79, 80]. These structures are similar to the GCs of secondary lymphoid organs and have been described in systemic lupus erythematosus, Hashimoto's thyroiditis, Graves' disease, rheumatoid arthritis, Sjögren's syndrome, multiple sclerosis, and type 1 diabetes [81–83]. The function and potential pathogenic role of ectopically formed lymphoid structures within inflamed tissues remains unclear. However, plasma cells residing within the ectopic GCs secrete autoantibodies [84], making it plausible that ectopic GCs have a role in the maintenance of immune pathology [85, 86]. 

Recent research has demonstrated that B cells are also involved in the inhibition of inflammatory immune responses, a function carried out by a subpopulation of B cells fittingly named regulatory B cells or Bregs.

## 3. IL-10 Secreting B Cells and Regulatory B Cells

A role of B cells in the inhibitory regulation of immune responses was initially suggested in autoimmune mice, where absence of B cells led to increased inflammation [87–89]. Transfer of wild-type B cells, but not IL10-negative B cells, reversed the inflammatory response [90], and IL-10 producing B cells were shown to suppress inflammation in mouse models of autoimmune diseases [91–93]. The significance of this anti-inflammatory cytokine was further supported by the finding that IL-10-deficient mice showed more severe disease accompanied with an increase in Th1 cytokine levels [88, 94, 95] and lower levels of regulatory T cells [96]. IL-10 is secreted by monocytes, Th2 T cells, regulatory T cells, and a rare subset of B cells. These IL-10 secreting B cells [97–100] can suppress CD4+ T cell responses and prevent autoimmune disease in mouse models and have been fittingly named regulatory B cells or Bregs [98–100]. The involvement of Bregs in human disease was first suggested by the observation that B-cell depletion can exacerbate Th-1-mediated autoimmune conditions such as ulcerative colitis [101] and psoriasis [102], and IL-10 producing B cells have been identified in humans [65]. For detailed discussions of Bregs please refer to other excellent reviews [99, 103].

## 4. B-Cell Tolerance

B-cell tolerance is established at multiple checkpoints throughout B-cell development, both in the bone marrow and the periphery. It has been estimated that 50% to 75% of newly produced human B cells are autoreactive and must be eliminated by tolerance mechanisms [104]. Induction of B-cell tolerance starts in the bone marrow. The major elimination mechanisms are receptor editing, clonal deletion, and anergy [105–107]. Defects in this early tolerance induction have been observed in subjects with rheumatoid arthritis, systemic lupus erythematosus, and type 1 diabetes [53, 108–110].

Once autoreactive B cells are removed, the immature B cells leave the bone marrow and migrate to the spleen, where they may encounter autoantigen not present in the bone marrow. B cells with high avidity to autoantigen are deleted, while low-avidity or very-low avidity interactions lead to anergy or ignorance, respectively [111]. 

An encounter with true foreign antigen triggers the migration of the B cell to the T-cell zone of GCs, and activation by antigen-specific CD4+ T cells. During the ensuing rapid proliferation phase B cells undergo somatic hypermutation predominantly of the variable regions of their immunoglobulins. Only those B cells that express antibodies with increased affinity are selected to survive and exit the GC as antibody producing plasma cells or memory cells (for details see [112]). 

### 4.1. Loss of Tolerance

Any of the above-discussed tolerance checkpoints can be faulted by genetic mutations allowing autoreactive B cells to survive. Some of these mutations have been identified in mouse models of autoimmune diseases with parallel findings in human disease.

1. Faulty negative selection at the immature B cells stage: NZM2410 mice spontaneously develop severe lupus nephritis at an early age. These mice carry the lupus susceptibility locus Sle1 containing at least three subloci, Sle1a, Sle1b, and Sle1c, involved in B-cell tolerance and activation of CD4+ T cells [113]. Using Sle1 congenic C57Bl6 mice, Kumar and colleagues [114] showed that mutations located within the Sle1 induced loss of B-cell tolerance through impaired negative selection of autoreactive B cells at the immature B-cell stage.
2. Increased B-cell signaling by overexpression of BCR signal-enhancing molecules or deficiency of molecules inhibiting BCR signaling: CD19 is a B-cell surface molecule that decreases the threshold for BCR stimulation. Hyperexpression of CD19 in mice led to increased levels of serum antibodies and increased B-cell activation, while the loss of CD19 reversed these phenotypes [115–119]. Deficiency of molecules that inhibit BCR-signaling, such as SHP-1 [120], Lyn [121], or Fc*γ*RIIB [122], causes increased B-cell signaling and initiates development of systemic autoimmunity in mice. The inhibitory Fc*γ*RIIB is expressed on B cells, where it regulates activating BCR signals. Lack of Fc*γ*RIIB expression leads to autoimmunity and autoimmune diseases [122–124]. The importance of Fc*γ*RIIB in human autoimmunity is exemplified by the finding that B cells from patients with lupus express lower levels of Fc*γ*RIIB on their surface due to polymorphisms in their Fc*γ*RIIB promoter [125], or the receptor itself [126, 127].
3. Generation of autoreactive immunoglobulins during somatic hypermutation: during affinity maturation the massive somatic hypermutations can also cause the inadvertent development of autoreactive immunoglobulins. While normally the resulting autoimmune B cells may either not receive necessary survival signals [128] or be eliminated, they accumulate in autoimmune diseases.
4. Increased survival of autoreactive B cells: B-cell activation factor (BAFF) is a B-cell survival factor and overexpression of BAFF in transgenic mice led to an expansion of peripheral B cells with higher autoantibody levels and the development of a lupus-like disease in the animals [28]. Elevated serum levels of BAFF have been found in patients with rheumatoid arthritis, systemic lupus erythematosus, and primary Sjörgren's syndrome [129–131]. These observations make BAFF a potential target for therapy [132, 133]. Indeed neutralization of BAFF was shown to be associated with loss of mature B cells [134] and reduced symptoms of autoimmune diseases in animal models [135, 136].

In the following the role of B cells in autoimmune diseases will be discussed in the context of systemic lupus erythematosus, rheumatoid arthritis, and type 1 diabetes. Systemic lupus erythematosus is a classic B-cell-mediated autoimmune disease, while rheumatoid arthritis and type 1 diabetes were initially considered to be predominantly T cell mediated. However recent studies suggest a role of B cells in the pathogenesis of these autoimmune diseases, as will be discussed in detail below.

*Systemic Lupus Erythematosus (SLE)* is a complex autoimmune disease, characterized by hyperglobulinemia, immune complex deposition, and end organ damage. B cells have been identified as major contributors to SLE, and B-cell depletion in SLE animal models abrogated the development of disease [54, 137]. Indeed, generalized B-cell hyperactivity has been documented in several murine models of lupus [138] and is also evident in patients with lupus [139, 140], where the number of B cells at all stages of activation is increased during active disease [141]. Both the decrease in proapoptotic genes and the increase in prosurvival gene expression have been suggested to cause this prolonged half-life of B cells in SLE (see also above). 

A pathogenic role of autoantibodies in SLE is supported by the observation that the passive transfer of anti-DNA antibodies induces distinct features of lupus nephritis in healthy animals [142, 143]. Autoantibodies in SLE contribute to end organ damage in glomerulonephritis (glomerular antibodies and anti-DNA antibodies) [144–146], congenital heart block (anti-Ro antibodies) [147], and thrombosis (anticardiolipin antibodies) [148]. Other autoantibodies are directed to diverse self-molecules, most notably antinuclear antibodies directed to double stranded DNA (dsDNA) [149], and small nuclear ribonucleoprotein (snRNP). However, B cells also have antibody-independent effects on the SLE pathogenesis. These functions include antigen presentation, costimulation of T cells, and secretion of proinflammatory cytokines. This role was evaluated in a set of experiments conducted by Chan and colleagues, where B cells in a SLE mouse model carried a mutation that prevented the secretion of antibodies [54]. Thus these animals had B cells but were devoid of circulating antibodies. Despite the absence of autoantibodies, the mice developed nephritis, indicating an antibody-independent effect of B cells. B-cell-deficient MRL/lpr mice remain disease-free and fail to develop activated CD8+ and CD4+ T cells found in B-cell-sufficient mice, a finding attributed to loss of B cell-CD4 T cell interactions [150].

The dual effect of IL-10 as a B-cell stimulator and inhibitor of T-cell activation is exemplified in SLE [151]. In mice models for SLE, IL-10 appears to exert mainly its above-discussed anti-inflammatory effect and IL-10-deficient mice develop a more severe disease with increased proinflammatory cytokine levels [152], while transfer of IL-10 producing B cells induced the expansion of regulatory T cells [96]. However, in human SLE IL-10 promotes disease, IL-10 serum levels are significantly elevated and correlate with disease activity [153] and IL-10 induced a significant increase of anti-DNA antibody secretion in cultured PBMCs from SLE patients [154]. This antibody secretion was significantly reduced in the presence of neutralizing IL-10-specific antibodies [155] and treatment with IL-10-specific monoclonal antibodies led to marked improvement in participants of a small clinical trial [156]. The protective effect of IL-10 in mice appears to be mediated through T-cell regulation, as IL-10 overexpression in a mouse model for lupus resulted in reduced T-cell activation, while B-cell phenotypes remained unaffected [151]. In SLE patients immune cells that normally suppress B-cell activation are defective and do not counteract the IL-10-mediated stimulation of B cells resulting in the subsequent secretion of autoantibodies [157].

*Rheumatoid Arthritis (RA)* is a chronic inflammation of the joint capsule (synovium) and synovial membranes, associated with proliferation of synovial fibroblasts and macrophages, leading eventually to cartilage injury and bone erosion [158]. While T cells are a major component in the pathogenesis, several observations suggest that B cells are necessary for the development of the disease, as B-cell deficiency in RA animal models abrogates disease [159, 160], and autoimmune T cells alone are not sufficient to induce disease [161]. At least two mechanisms of B-cell involvement are currently considered: the production of autoantibodies and antigen presentation. Autoantibodies in patients with RA typically target several autoantigens, including rheumatoid factor (RF), type II collagen (CII), and citrullinated proteins (ACPA). A model for the pathological role of RA-associated autoantibodies will be discussed for autoantibodies directed to CII. These autoantibodies are found in ~70% of patients with early RA [162–164] both in their serum and synovial fluids. A pathogenic role of CII-specific antibodies was indicated in an animal model termed collagen-induced arthritis (CIA), where immunization of animals with CII induced the development of CII antibodies [165] and triggered arthritic symptoms [166–168]. Moreover, arthritic symptoms were also observed after passive transfer of CII-reactive serum obtained from CIA animals [169], patients with RA [170], or monoclonal antibodies specific to CII [165, 171] to healthy recipient animals, further supporting a pathological role of CII antibodies. CII autoantibodies are thought to mediate the formation of immune complexes in the joint, followed by complement activation and inflammatory cell recruitment. After Fc*γ*R ligation, the activated cells secrete proinflammatory cytokines, further activating an immune reaction consisting of synovial macrophages and infiltrating mononuclear cells with the eventual release of tissue-degrading enzymes that cause cartilage damage [172]. CII autoantibodies may also have a direct pathogenic function, which occurs in the absence of inflammatory mediators [173]. Here the antibodies modify the synthesis of collagen fibrils effecting cartilage synthesis and stability [174–176], possibly through steric hindrance of collagen epitopes that are important for the formation of collagen fibrils [177–179].

*Type 1 Diabetes (T1D)* is an organ specific autoimmune disease, characterized by the destruction of the insulin-producing beta cells in the pancreas. During progression towards T1D the pancreatic islets are infiltrated by mononuclear cells consisting of CD4+ and CD8+ T cells, B cells, macrophages, and dendritic cells [180, 181]. Both CD4+ and CD8+ T cells contribute to the ultimate attack on the beta cells [182], but in recent years the pathogenic role of B cells is beginning to emerge [183, 184]. A major hallmark of the autoimmunity leading to T1D is the presence of autoantibodies to beta cell antigens. At the time of clinical diagnosis more than 90% of patients present at least one of the T1D-associated autoantibodies [185]. The four beta cell antigens most frequently targeted by autoantibodies are insulin [186], the smaller isoform of glutamate decarboxylase (GAD65) [187], protein-tyrosine-phosphatase-like protein IA-2 [188], and the zinc transporter 8 (ZnT8) [189]. These autoantigens are also targeted by autoreactive T cells, suggesting a collaborative interaction between T and B cells [190]. No direct pathogenic role has been assigned to these autoantibodies and they are generally viewed as markers only. However a potential role of GAD65Ab in enhanced antigen uptake has been suggested [191]. Stimulation of GAD65-specific T-cell clones with human recombinant GAD65 was tested in the presence of sera obtained from GAD65Ab-positive T1D patients and GAD65Ab-negative T1D patients. Only sera from GAD65Ab-positive patients significantly enhanced T-cell stimulation. Moreover, this effect was inhibited by monoclonal antibodies to the FcR, suggesting Fc-mediated uptake of GAD65 complexed with GAD65Ab as the underlying mechanism.

However, the major mechanism by which B cells contribute to T1D development is the antibody-independent presentation of beta cell antigens [190, 192, 193]. Nonobese diabetic (NOD) mice deficient of mature B cells do not develop T1D [193–199]. In the absence of B cells, NOD mice showed significantly lower numbers of CD4+ and CD8+ T cells in their insulitic lesions [62, 195, 198–200], suggesting a role of B cells in the activation of autoreactive T cells. The function of B cells as APCs was illustrated in NOD mice whose B cells were rendered MHC class II deficient [201]. Although these animals retained their ability to present antigen via dendritic cells and macrophages, they were protected from diabetes development. However, the presence of insulitis in B-cell-deficient mice [62] and the report of at least one B-cell-deficient T1D patient [202] indicate that B cells may not be absolutely essential for the development of T1D and can be substituted by other APCs. As discussed above, B cell can focus the immune response towards a specific antigen. NOD mice that expressed only B cells specific to an irrelevant antigen (Hen Egg Lysosome) did not develop an autoantigen-specific T-cell response and remained healthy, indicating that only autoantigen-specific B cells enhance the development of T1D in the NOD mouse [203]. We will discuss the role of autoantigen-specific B cells exemplified by GAD65-specific B cells. Although GAD65 levels in murine pancreatic beta cells are very low, it is a major autoantigen in the pathogenesis of T1D in the NOD mouse [204]. GAD65-specific T cells have been demonstrated in both T1D patients and the NOD mouse [205–209]. Adoptive transfer of GAD65-reactive T cells isolated from NOD mice caused recipient animals to develop T1D [207, 210], supporting the concept of diabetogenic GAD65-specific T cells in the pathogenesis of T1D. Importantly, the development of these GAD65-specific T cells depends on the presence of B cells [190, 192, 203]. The finding that reconstitution of B-cell-depleted NOD mice with B cells reinstated T1D only if the repopulating B cells were primed with GAD65 [190] suggests that B-cell-mediated presentation of GAD65 stimulates GAD65-reactive T effector cells to target pancreatic beta cells. It is however not only the antigen specificity, but also the epitope specificity of the B cells that affects the T-cell response. GAD65-specific B-cell hybridomas with different epitope specificities were tested for their capacity to stimulate GAD65-specific T-cell clones. Those T-cell clones whose epitope lays outside of the BCR epitope showed increased T-cell responses, while T-cell clones whose epitope lays inside the BCR epitope showed suppressed responses, suggesting that the BCR epitope specificity can promote the presentation of some T-cell determinants, while suppressing that of others [211, 212]. 

Based on the promising results of B-cell depletion in the prevention of T1D in NOD mice, the effect of B-cell depletion on human T1D was tested in a phase II multicenter clinical trial on newly diagnosed human T1D patients [213]. One year after treatment a delay in the loss of beta cell function as shown by the preservation of C-peptide was demonstrated. Moreover, patients required less insulin and had better overall blood glucose control. These results confirm that B cells contribute also to human T1D. 

**Figure 2:**
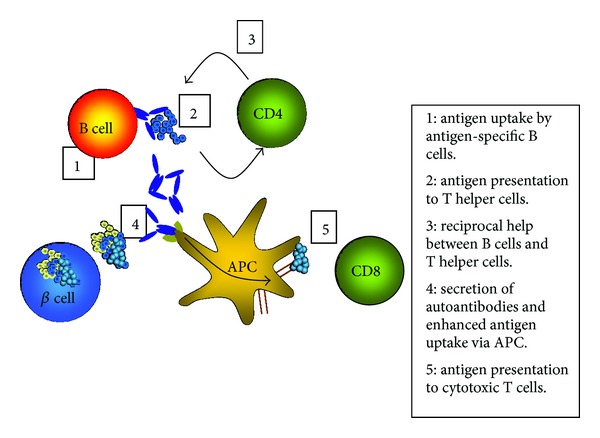
Model of pathogenic function of B cells in type 1 diabetes. Islet cell antigen released from the pancreatic beta cells is being taken up at low antigen concentrations by antigen-specific B cells, which present the antigen determinants to CD4+ T cells. T cells provide help to the B cells to eventually differentiate into antibody secreting plasma cells. Autoantibodies can now bind to the autoantigen and the resulting autoantibody/autoantigen complexes are efficiently taken up via FcR present on other APCs. This enhanced autoantigen uptake and presentation finally activates cytotoxic CD8+ T cells, which carry out the killing of the beta cells.

Gathering the current understanding of B cells in T1D, the following mechanisms have been suggested (Figure 2). Beta cell antigen is taken up via BCR by antigen-specific B cells (1) and presented on MHC class II molecules to CD4+ T cells (2). Activated CD4+ T cells provide help to B cells (3). B cells differentiate to plasma cells and secrete autoantibodies (4). These autoantibodies form autoantigen-autoantibody complexes that bind to the Fc*γ*R on other APCs (5). This enhanced antigen presentation eventually triggers both natural killer cells and CD8+ T cells to attack the pancreatic beta cell.

## 5. B-Cell Depletion

The growing understanding that B cells play a pathological role also in autoimmune diseases that are traditionally viewed as T cell mediated led to B-cell depletion treatment not only in diseases that are clearly B cell dominated, but also in autoimmune diseases that are traditionally viewed as T cell mediated, such as T1D.

B-cell depletion can target a number of different B-cell molecules, either with the goal of B-cell elimination, or the suppression of survival. Four major classes of B-cell targeting drugs have been evaluated for the treatment of autoimmune diseases: neutralization of survival factors BAFF and APRIL [214], killing of B cells using monoclonal antibodies directed to CD19, CD20, and CD22 [215–217], induction of apoptosis using reagents targeting the BCR itself or BCR associated transmembrane signaling proteins such as CD79 [193, 218], and ablation of the formation of ectopic GCs by antibodies against lymphotoxin-*β* receptor (LT*β*R) [219].

**Figure 3:**
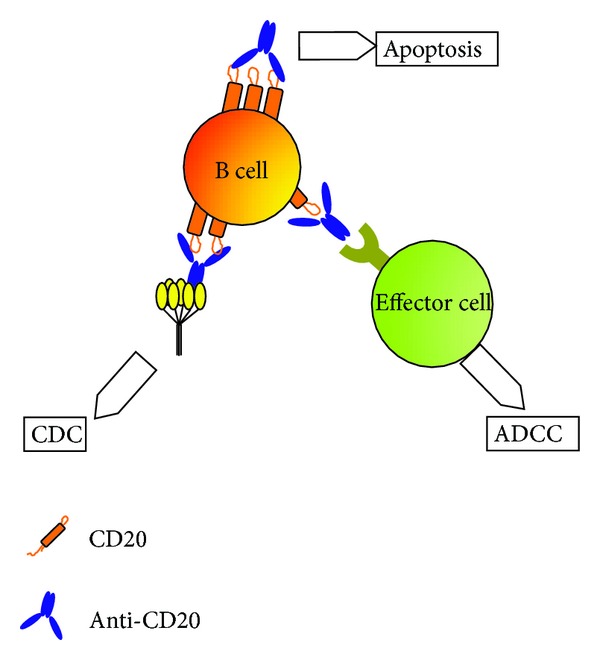
B-cell depletion with CD20 (Rituximab). Anti-CD20 mAb can direct the killing of B cells by antibody-dependent cytotoxicity (ADCC), complement-dependent cytotoxicity (CDC), or apoptosis. ADCC is triggered by the interaction between the Fc region of the antibody and the FcR on effector cells of the immune system. In CDC the Fc region is bound by the complement component C1q, which triggers a proteolytic cascade. Apoptosis occurs when CD20 molecules are cross-linked by anti-CD20 mAb in lipid rafts and activate signaling pathways leading to cell death.

B-cell depletion for treatment of human autoimmune diseases is often accomplished through antibodies targeting the surface molecule CD20 (e.g., Rituximab and Ofatumumab). Treatment with these antibodies depletes B cells by a combination of antibody-mediated cellular cytotoxicity (ADCC), complement-dependent cytotoxicity (CDC), and antibody-triggered apoptosis [220] (Figure 3). The CD20 density on B cells appears to be important for CDC, since it is highly correlated with CDC [221]. CD20mAb/CD20 immune complexes aggregate in microdomains, where the antibodies' Fc regions are bound by C1q, leading to complement activation [222]. CD20 may also act as a signaling molecule to trigger apoptosis when engaged with CD20mAb [223, 224]. 

B-cell depletion using Rituximab has been used for the treatment of a number of autoimmune and chronic inflammatory diseases [213, 225, 226]. Rituximab treatment results in nearly undetectable circulating B-cell levels one month after therapy and B cell counts remain low for 6–12 months [227]. Because the drug targets B cells expressing surface CD20, mature and memory CD20+CD27+ B cells in blood and primary lymphoid organs are effectively depleted, while long-lived plasma cells are not directly depleted [228], and Rituximab treatment appears not to affect circulating IgG levels [229], while reducing circulating IgM levels [230]. This effect of Rituximab is illustrated by the observation that immunization within the first 9 months after Rituximab treatment results in significantly reduced antibody responses, which develop from IgM-positive B cells [231, 232]. It is therefore of interest that for some autoimmune diseases B-cell depletion was reported to be associated with a decrease in IgG autoantibody titers [77] and specific depletion of autoreactive B cells by CD20mAb was demonstrated in mice [233]. As bone marrow stem cells and early B-cell precursors (pro-B cells) do not express CD20 [234], the new naïve B cells repopulate the B-cell compartment once the drug has cleared the system, allowing the immune response to return to normal. Disease relapses in about 50% of patients either at the time that B-cell numbers increase to pretreatment levels or within 3 months, while in other cases clinical relapse can be delayed for years [235]. Additional Rituximab courses can induce subsequent remission [236]. Multiple Rituximab courses are often associated with progressive decrease in circulating IgM [237] and IgG levels [238].

The antibody-independent effect of Rituximab treatment may be due to the elimination of B cells as APC and subsequent reduced stimulation of T cells [239, 240]. However, not all CD20+ B cells are equally affected by Rituximab treatment. B cells located in the peritoneal cavity are surprisingly resistant to depletion [241]. While these B cells express normal CD20 densities and are bound by CD20mAb, only about 50% of these cells are depleted. These location-dependent sensitivities to CD20mAb-mediated depletion could have significant consequences for therapy and may be the reason of the heterogeneity of results in human clinical trials. Other factors such as gender, age, and weight [242] and immunological profile [243] affect the outcome of Rituximab treatment. The major side effect of B-cell depletion is the risk of severe infections, which needs to be taken into consideration when evaluating the risks and benefits of B-cell depletion [244, 245]. 

In summary, B-cell depletion offers a promising therapy for the treatment of a variety of autoimmune diseases. The treatment is usually well tolerated; however, adverse events include infusion reactions, infections, and hypogammaglobulinemia. 

## 6. Conclusions and Future Directions

The traditional concept of T-cell-mediated and autoantibody-mediated autoimmune diseases needs to be adjusted to reflect the interaction of different immune cells in autoimmune pathogenesis. The recognition of the contribution of B cells in the pathogenesis of autoimmune diseases, which are traditionally viewed as T cell mediated, led to promising immune-modulating therapies.

Global B-cell depletion eliminates both protective and pathogenic B cells. The success of B-cell depletion is therefore dictated by the extent of depletion of protective versus pathogenic B cells. The hopes that B-cell depletion would allow the restoration of immunological tolerance with long-term remission were not fulfilled, as is evident from the recurrence of autoimmune disease after the B-cell compartment is replenished. Selective depletion of antigen-specific B cells may provide an alternative to global B-cell depletion. This approach has the additional advantage that unlike Rituximab treatment it may also eliminate CD20-long-lived autoreactive plasma cells. 

Several mechanisms are currently investigated in different *in vitro* and *in vivo* models of autoimmune diseases, a few of which will be discussed here.

Autoantigens can be fused to the IgG1 Fc domain to activate complement and FcR-dependent effector cell responses. This approach has been successfully evaluated *in vitro* and *in vivo* for the treatment of multiple sclerosis by autoantigen fused to Fc, which induced the effective and specific effector lysis of autoantigen-specific B cells [246]. An inhibitory B-cell signal can be induced by cross-linking of the autoantigen-specific BCR with the inhibitory Fc*γ*RIIb. Autoantigen fused to an Fc*γ*RIIb-binding mAb successfully reduced autoantibody levels and disease symptoms in lupus-prone MRL/lpr mice [247–249]. Autoantigen can also be coupled to an antibody specific to complement receptor 1 (CR1). CR1 negatively regulates the proliferation and differentiation of activated B cells after binding C3b [250]. In a small clinical trial SLE patients treated with dsDNA coupled to a CR1-specific monoclonal antibody showed a significant reduction of dsDNA autoantibody titers [251]. In an early study, Blank et al. employed anti-idiotypic antibodies directed to a pathogenic anti-DNA idiotype. Administration of this anti-idiotypic antibody alone or coupled to the cytotoxin saporin induced a significant reduction in anti-DNA antibody titer and diminished clinical manifestation in lupus-prone mice [252]. In a similar approach we demonstrated that GAD65Ab-specific anti-idiotypic antibodies protected NOD mice from development of T1D [253]. In addition to the direct elimination of antigen-specific B cells, autoantigen-fusion proteins can also bind pathogenic autoantibodies and route them to clearance. 

Recently Bollmann proposed the targeted elimination of autoantigen-specific B cells using artificial antigens linked to magnetic nanoparticles. Here the autoantigen-specific B cells would be removed in an extracorporeal filtration method in an attempt to suppress or cure the autoimmune response [254].

The feasibility of these specific B-cell depletion approaches needs to be further evaluated; however, they offer new therapeutic options for the treatment of autoimmune diseases.

## Figures and Tables

**Table 1:** Examples for receptor autoantibodies.

Targeted receptor	Mechanism	Associated disease	References
Endothelial receptor type A (ET_A_R)	Activation	Pulmonary arterial hypertension (PAH)	[1]
Angiotensin II receptor (AT_1_R), ET_A_R	Activation	Systemic sclerosis	[2]
AT_1_R	Activating	Preeclampsia	[3–5]
*α*_1_-adrenergic receptors (*α*_1_-ARs)	Activating	Refractory hypertension	[3, 6, 7]
*β*_1_-adrenergic receptor	Activation	Dilated cardiomyopathy (DCM), Chagas' disease	[8, 9]
N-methyl-D-aspartate receptor (NMDAR)	Activation	SLE	[10]
Glutamate receptor	Activation	SLE	[11]
Insulin receptor	Inhibition	Autoimmune hypoglycemia	[12]
Muscarinic type 3 receptor	Internalization	Sjögren's syndrome	[13]
NMDAR	Internalization	Anti-NMDA receptor encephalitis	[14]

## Abbreviations

AChR: ACh receptor
ACPA: Citrullinated proteins
ADCC: Antibody-dependent-cell-mediated cytotoxicity
APCs: Antigen presenting cells
BAFF: B-cell activation factor
BCR: B-cell receptors
Bregs: Regulatory B cells
CII: Type II collagen
CDC: Complement-dependent cytotoxicity
FcR: Fc receptor
Fc*γ*R: Fc gamma receptor
FDCs: Follicular dendritic cells
GAD65: 65 kD isoform of glutamate decarboxylase
GC: Germinal centers
IA-2: Protein-tyrosine-phosphatase-like protein
IFN-*γ*: Interferon-gamma
LT*α*_1_*β*_2_: Membrane-bound lymphotoxin *α*_1_ *β*_2_
LT*β*R: Lymphotoxin-*β* receptor
MHC: Major histocompatibility complex
mIgM: Membrane IgM
NOD: Nonobese diabetic
OVA: Ovalbumin
RA: Rheumatoid arthritis
RF: Rheumatoid factor
SLE: Systemic lupus erythematosus
T1D: Type 1 diabetes
TPO: Thyroid peroxidase
TSH: Thyroid stimulating hormone
ZnT8: Zinc transporter 8.
